# Hernia reduction following laparotomy using small stitch abdominal wall closure with and without mesh augmentation (the HULC trial): study protocol for a randomized controlled trial

**DOI:** 10.1186/s13063-019-3921-3

**Published:** 2019-12-16

**Authors:** Patrick Heger, Manuel Feißt, Johannes Krisam, Christina Klose, Colette Dörr-Harim, Solveig Tenckhoff, Markus W. Büchler, Markus K. Diener, André L. Mihaljevic

**Affiliations:** 1 Department of General, Visceral and Transplantation Surgery, University Hospital Heidelberg, Im Neuenheimer Feld 110, 69120 Heidelberg, Germany; 2 The Study Center of the German Surgical Society (SDGC), University Hospital Heidelberg, Im Neuenheimer Feld 130.3, 69120 Heidelberg, Germany; 3 Institute of Medical Biometry and Informatics (IMBI), University of Heidelberg, Im Neuenheimer Feld 130.3, 69120 Heidelberg, Germany

**Keywords:** Abdominal wall closure, Small stitches technique, Onlay mesh augmentation, Incisional hernia, Surgical site infection, Laparotomy, Randomized controlled trial

## Abstract

**Background:**

Incisional hernias are among the most frequent complications following abdominal surgery and cause substantial morbidity, impaired health-related quality of life and costs. Despite improvements in abdominal wall closure techniques, the risk for developing an incisional hernia is reported to be between 10 and 30% following midline laparotomies. There have been two recent innovations with promising results to reduce hernia risks, namely the small stitches technique and the placement of a prophylactic mesh. So far, these two techniques have not been evaluated in combination.

**Methods:**

The HULC trial is a multicentre, randomized controlled, observer- and patient-blinded surgical effectiveness trial with two parallel study groups. A total of 812 patients scheduled for elective abdominal surgery via a midline laparotomy will be randomized in 12 centres after informed consent. Patients will be randomly assigned to the control group receiving closure of the midline incision with a slowly absorbable monofilament suture in the small stitches technique or to the intervention group, who will receive a small stitches closure followed by augmentation with a light-weight polypropylene mesh in the onlay technique. The primary endpoint will be the occurrence of incisional hernias, as defined by the European Hernia Society, within 24 months after surgery. Further perioperative parameters, as well as patient-reported outcomes, will be analysed as secondary outcomes.

**Discussion:**

The HULC trial will address the yet unanswered question of whether a combination of small stitched fascial closure and onlay mesh augmentation after elective midline laparotomies reduces the risk of incisional hernias. The HULC trial marks the logical and innovative next step in the development of a safe abdominal closure technique.

**Trial registration:**

German Clinical Trials Register, DRKS00017517. Registered on 24th June 2019.

## Background

Incisional hernias (IHs) are among the most frequent complications following open abdominal surgery [1]. IHs cause substantial morbidity and costs and reduce health-related quality of life (HRQoL) [2]. In recent years several randomized controlled trials (RCTs) have been conducted comparing different techniques of abdominal wall closure. These trials have shown convincingly that abdominal wall closure with a continuous running suture is superior to interrupted suture techniques, at least in the elective setting [3–6]. Similarly, slowly absorbable suture material creates less IHs than rapidly absorbable sutures [3, 4, 7, 8]. These results were summarized in a meta-analysis [9]. Despite these advances, IH risks of 10–30% are regularly reported in RCTs following abdominal wall closure [3–6, 10] and increase to 36% in certain subgroups [11–14]. Furthermore, IH risks increase with extended time of follow-up [15].

Since the INLINE meta-analysis in 2010 [9], there have been two recent innovations in the field of abdominal wall closure aiming at a reduction of incisional hernias: the small stitches technique (SST) [10, 16] and prophylactic mesh placement [17, 18]. SST abdominal closure using a slowly absorbable suture and an increased suture-length to wound-length ratio of ≥4 significantly reduced IH in a pseudorandomized trial by Millbourn et al. [10]. A recent multicentre RCT has verified the superiority of this technique in terms of reduced IH frequency in comparison to standard abdominal wall closure [1]. However, even under these optimized conditions, 13% of patients developed an IH after 12 months [1].

Regarding prophylactic mesh placement, several RCTs have been performed in specific subsets of patients, with encouraging results [11, 19–23]. A meta-analysis confirmed a significantly lower risk of IHs in the mesh group [17]. Most importantly, other wound complications like surgical site infections (SSIs) were not increased in the mesh groups [17]. This is in line with the results of a multicentre RCT comparing prophylactic mesh placement to primary closure (PRIMA trial) that confirmed the superiority of prophylactic mesh placement [18, 24]. PRIMA compared onlay mesh augmentation (OMA) or sublay mesh augmentation (SMA) vs. primary suture closure of the abdominal incision in high-risk patients and identified significantly fewer patients with an IH in the OMA group (13%) than in the primary suture group (30%) after 2 years (odds ratio [OR] 0.37; 95% confidence interval [CI] 0.27 to 0.77; *p* = 0.0016). Comparing the SMA group with primary suture, the results just failed statistical significance (SMA vs. primary suture 18% vs. 30%; OR 0.55; 95% -CI 0.30 to 1.00; *p* = 0.05).

However, in none of these trials was prophylactic mesh placement combined with SST. A combination of these two techniques, which showed effectiveness as single interventions, is considered to be the logical consequence and may result in an additional reduction of IH formation.

## Methods/design

### Trial rationale

The objective of the HULC trial is to investigate whether prophylactic OMA in addition to abdominal wall closure in SST reduces the risk of IH formation in patients undergoing elective midline laparotomy compared to SST alone.

### Trial design

HULC is a multicentre, randomized controlled, observer- and patient-blinded surgical effectiveness trial with two parallel study groups.

### Patients and trial centres

To enrol the required number of patients in the planned recruitment period, 12 trial sites of the Clinical Trials Network of the German Surgical Society (CHIR-*Net*) will participate in this trial (www.chir-net.de). These 12 centres will be high-volume centres committing to include at least 50 patients each. To improve recruitment at all centres, brochures and flyers for patients including information about the trial will be available.

### Patient inclusion criteria

All patients scheduled for elective clean or clean-contaminated [25] abdominal surgery as defined by the Centers for Disease Control and Prevention (CDC) via a midline laparotomy for any indication will be screened consecutively for eligibility and will be informed about the HULC trial. All subjects must be able to understand the nature and extent of the trial, and only adult patients (> 18 years of age) with a life expectancy of at least 2 years who provide written informed consent will be included in the trial. The participant informed consent form includes general information about the trial (indication, clinical data about incisional hernias) as well as details about the experimental and control intervention, randomization, benefits and risks of participation in the trial and trial visits. Furthermore, it includes general study information like the voluntariness of participation, possibility of termination of the trial, organization and financing of the trial, data protection and important contact details for further questions. As the intervention of the trial includes two well-established surgical standard therapies for abdominal wall closure, there is no increased risk of any harm to be expected through participation in the trial. Thus, no additional compensation for any postoperative complications or harm is planned. Patients will be insured against travel accidents for their follow-up visits.

### Patient exclusion criteria

Patients with planned re-laparotomy via the midline incision within 2 years after trial intervention, midline laparotomy within the last 60 days prior to trial intervention or previous IHs or fascial dehiscences will be excluded from the trial. Moreover, patients with concurrent abdominal wall infections will not be included in the trial, in order to reduce the risk of SSI and potential mesh infections. Furthermore, patients with an American Society of Anesthesiologists (ASA) grade > 3 classification, pregnant or lactating women and patients who participate in another intervention trial with interference of the intervention and/or outcome of the HULC trial will be excluded.

### Patient withdrawal criteria

Patients are free to stop their trial participation at any time and without giving reasons for their decision. When a trial participant withdraws his/her informed consent, he/she is asked to decide whether his/her data captured so far may be analysed or if it should be discarded. In addition, if, in the surgeon’s opinion at the end of the operation, the trial intervention will be detrimental to the subject’s well-being, the trial participation can be stopped for this patient. In this case, the patient will not be randomized, and the reason for screening failure must be recorded in the screening log. All randomized patients, including those with premature trial termination, will be included in the final analysis.

### Control intervention

Patients in both groups will receive closure of the midline incision with a slowly absorbable monofilament suture (USP 2-0, PDS Plus, Ethicon, Somerville, NJ, USA) in SST as in previous trials [1, 10]. Tissue bites of 5 mm and intersuture spacing of 5 mm are applied exclusively to the fascia within the linea alba (omitting subcutaneous fat and muscle tissue). Suturing will be initiated at both ends of the median laparotomy towards the centre. An overlap of up to 2 cm may be created. Both sutures should be knotted independently. The suture-length to wound-length ratio (SL:WL) must be ≥4:1. The SL:WL ratio is recorded intraoperatively and is calculated as follows [16]: SL:WL = (*A*-(*B* + *C*)) / *D* (*A* = total length of suture used; *B* = length of suture remnants at starting knots; *C* = length of suture remnants at finishing knots; *D* = length of fascial incision (all in centimetres)).

### Experimental intervention

In the experimental group, but not in the control group, the abdominal wall closure is augmented with a light-weight polypropylene mesh in onlay technique (OMA). To this end an anterior plane will be created between the anterior rectus fascia and the subcutis. The mesh should overlap the fascial midline incision by 3–4 cm on all sides [18, 24] and must be fixed to the fascia tension-free with USP 2-0 Prolene single knots (Ethicon, Somerville, NJ, USA). The mesh material will be standardized and an Optilene® Mesh (B. Braun, Melsungen, Germany) will be used. The interventional procedure will prolong the operation by approximately 20 min.

The materials and surgical technique will be standardized. HULC will use the same materials and surgical technique as the previous PRIMA and STITCH trials [1, 24] to ensure comparability of results and to avoid potential bias.

Closure technique of the skin and the subcutaneous tissue will be the same in both groups and will be standardized to reduce dead space and seroma formation. The subcutaneous tissue should be closed with monofilament or polyfilament absorbable sutures. No subcutaneous drains should be placed. The subcutaneous sutures in the experimental group will include the mesh in the midline in order to reduce seroma formation, as the latter was increased in previous OMA trials without subcutaneous sutures [18, 24] but not in OMA trials with subcutaneous sutures [19]. The skin will be closed using staples.

### Assignment of intervention and randomization

In order to ensure equal distribution of patient characteristics, randomization will be used. Allocation of treatments will be performed using a web-based randomization tool (www.randomizer.at) by means of block-wise randomization. Randomization will be performed intraoperatively at the end of surgery, after closure of the fascia. This prevents potential bias by different intraoperative techniques. Before randomization, the surgeon needs to confirm a clean or clean-contaminated operation according to CDC definition [25]. Randomization will be stratified by centre and by IH risk (low-risk vs. high-risk patients, defined as patients with body mass index (BMI) ≥ 27 and/or those having surgery for abdominal aortic aneurysm). The surgeon who will perform the closing technique must be chosen before abdominal wall closure. As randomization is performed after closure of the facia in SST, surgeons call their respective clinical trial centre to have the online randomization performed and to receive the results by phone. Alternatively, the online randomization can be performed in the operating room by a third person, if the necessary equipment and internet access is available.

### Blinding

Patients, observers and data analysts will be blinded to the intervention in order to guarantee unbiased assessment of the primary outcome. The person performing randomization and the surgical team conducting the control/experimental intervention (“unblinded” study members) will be documented and will not be part of further outcome assessment. Moreover, neither the operation report nor the discharge letter will contain information regarding group allocation. In any case of an emergency including possible re-operation or a clinical situation that necessitates the knowledge of the trial group of the participant, patients can be unblinded.

### Other methods against bias

To minimize performance bias, the intervention will be standardized in both groups and the suture-length to wound-length ratio must be recorded intraoperatively and will be monitored. Furthermore, to minimize training effects, all participating surgeons must pass an obligatory eLearning tutorial demonstrating the SST before participation in the trial. Only surgeons having performed a minimum of 10 SST abdominal wall closures are allowed to perform interventions in the HULC trial. In addition, only centres committing to include at least 50 patients will participate in the trial.

### Primary endpoint

The primary outcome measure of the trial will be the occurrence of IHs within 24 months after surgery as defined by the European Hernia Society (EHS) [26]. Consequently, “any abdominal wall gap with or without a bulge in the area of a postoperative scar perceptible or palpable by clinical examination or imaging” is regarded as an IH. Occurrence of a burst abdomen will not be counted as a primary endpoint, but as a secondary endpoint by consensus [1, 3]. Follow-up time will be 24 months, as has been recommended by the EHS [26] since IH incidence increases over time [15]. Patients will be assessed for the primary endpoint at 6, 12 and 24 months after trial intervention. At these time points, patients will be examined by a clinician blinded for the trial intervention and by a radiologic examination performed by a blinded assessor. Radiologic exams allowed in the trial are sonography, computed tomography (CT) or magnetic resonance imaging (MRI) scans. In case of conflicting results between clinical and radiologic exams, the radiologic imaging is decisive to increase sensitivity [26]. If only one of the two examinations is performed (i.e. either clinical or imaging), the result of this assessment will be used for analysis. Possible results are listed in Table 1. As many patients included in this trial are expected to have an oncological indication for laparotomy and the included centres perform their oncological follow-up themselves, the loss to follow-up of patients is expected to be low. For patients who are unable or unwilling to attend the follow-up visits, a telephone follow-up is incorporated. The patient-reported outcome questionnaire developed by Jairam et al. [27] will be used, as it exhibits a high reliability. It will be used as a screening tool; i.e. patients who are suspected to have an IH based on the questionnaire might be convinced to attend an outpatient visit, even if they were reluctant to do so before.

**Table 1:** Definition of the primary endpoint for the HULC trial

Clinical exam result	Imaging result	Primary endpoint for HULC
Hernia	Hernia	Hernia
No hernia	Hernia	Hernia
Hernia	No hernia	No hernia
No hernia	No hernia	No hernia
Hernia	Missing	Hernia
No hernia	Missing	No hernia
Missing	Hernia	Hernia
Missing	No hernia	No hernia

### Primary estimand

In the recently released addendum to the ICH E9 guideline (draft version) [28], the estimands framework is recommended as a clear and transparent definition of “what needs to be estimated to address a specific scientific question of interest”. Such an estimand can be defined through the population of interest, variable of interest, specification of how intercurrent events are handled, and summary measure. The specification of how intercurrent events are handled is referred to here as intervention effect:

- *Population*. The population is defined as all patients fulfilling all the inclusion and none of the exclusion criteria.
- *Variable*. The variable is the occurrence of IHs as defined by the EHS within 24 months after intervention.
- *Intervention effect*. Possible intercurrent events and the strategies to handle them are as follows: missing values due to death, drop-out, loss to follow-up and re-laparotomy will be replaced by using multiple imputation. Since re-laparotomy changes the probability of occurrence of an IH, information of occurrence or non-occurrence of IH after re-laparotomy will not be considered for primary analysis. This represents a hypothetical strategy for the post-randomization events re-laparotomy, drop-out, loss to follow-up and death. Except for these events, other post-randomization events will not be considered, thus reflecting a treatment policy approach, which means that the effect of randomized treatment is estimated irrespectively of other post-randomization events not captured in the primary endpoint definition.
- *Summary measure*. The summary measure is the odds ratio (OR). The OR will be calculated by a two-level binary logistic regression analysis including the fixed factors treatment group and IH risk (low vs. high), the latter being deemed as the most important confounder by far and being also used for stratification in the randomization procedure, and the random factor centre. Confounding by other less important prognostic and predictive factors can assumed to be controlled by the randomized study design. The model will be fitted using a variance-components covariance matrix. The level of significance is set to 5% (two-sided). The *p* value for judging the primary hypothesis will result from the two-level binary logistic regression model, where the coefficient of the factor treatment effect is tested against zero using the Wald test.

Additionally, sensitivity and supplementary estimands will be considered, but are not described in further detail in this publication.

### Key secondary endpoints

The secondary measurements chosen in the HULC trial have been proposed by international guidelines [26]. For an adequate evaluation of the secondary endpoints, follow-up visits on postoperative days 5 to 7, 10 to 14 and 30 to 35 will be performed (see Table 2) in addition to the follow-up visits for the primary endpoint described above (at 6, 12 and 24 months postoperatively). The key secondary endpoints are as follows:

1. Risk of superficial and deep surgical site infections (SSIs) within 1 year in both groups [25]. SSIs will be assessed by clinical examination as defined by the CDC [25]. Organ-space SSIs are excluded in this measurement as they are independent of abdominal wall closure technique, but rather depend on the underlying surgery. Consequently, organ-space SSIs will be recorded in the overall complication rate and as serious adverse events (SAEs) if applicable. Follow-up for SSI is 1 year in line with CDC guidelines, as patients in the experimental group undergo implantation of alloplastic material (mesh).
2. Postoperative 30-day morbidity. Complications will be recorded and classified according to the Dindo-Clavien classification [30].
3. Occurrence of non-infectious wound complications (hematoma, seroma) within 30 days. Seroma is defined as a collection of serous fluid in a dead space, which can either be in situ or leaking through a wound. Hematoma is defined as an accumulation of blood in the wound area, which warrants (bedside) surgical exploration and intervention.
4. Occurrence of postoperative burst abdomen within 30 days. Postoperative burst abdomen will be defined as missing continuity of the fascia in combination with wound dehiscence with consecutive re-operation.
5. Postoperative wound pain at rest and during movement. Assessment will be performed using the well-established numeric pain rating scale. Pain is an important patient-reported outcome measure and is influenced by hernia occurrence and by surgery. Pain will be assessed at visits 3–5 (see Table 2), and the mean pain will be compared between the groups at these time points.
6. HRQoL measured with the Short Form Health Survey (SF-36) and EuroQoL five dimensions (EQ-5D) questionnaires. As HRQoL is an important patient-reported outcome measure and is influenced by hernia occurrence and by surgery [2], it will be recorded both preoperatively (visit 1) and during follow-up (visits 6–8) (see Table 2). The median HRQoL will be compared at these time points between the groups and also in terms of the change from baseline.
7. Length of primary hospital stay in days from index operation.

**Table 2:** Timetable of the trial according to Standard Protocol Items: Recommendations for Interventional Trials (SPIRIT) guidelines [29]

Activity	Visit 1 (screening)	Visit 2 (surgery, randomization)	Visits 3–5 (PODs 5–7, 10–14, 30–35)	Visits 6–8 (postoperative months 6, 12, 24)
Inclusion/exclusion criteria	X			
Informed consent	X			
Medical history	X			
Clinical examination	X		X	X
Surgery		X		
Randomization		X		
Incisional hernia assessment^a^				X
Assessment of SSI^b^			X	X (not at 24 months)^f^
Assessment of postoperative morbidity^c^			X	
Assessment of non-infectious wound complications			X	X
Assessment of burst abdomen			X	
Quality of life assessment^d^	X			X
Length of hospital stay			X	
Assessment of wound pain^e^			X	
Assessment of re-operations			X	X
Assessment of SAE		X	X	X

*POD* postoperative day, *SAE* serious adverse event, *SSI* surgical site infection

^a^Via blinded assessor: clinical and radiologic assessment

^b^Via blinded assessor according to CDC Definition [25]

^c^According to Dindo-Clavien

^d^According to SF-36 and EQ-5D questionnaires

^e^Using a numeric pain rating scale (NRS 1–10)

^f^As defined by the Centers for Disease Control CDC: “follow-up should be 30 days after the operation if no implant is left in place or 1 year if implant is in place” [25]

### Patient timeline and trial visits

Patients scheduled for elective abdominal surgery via a midline incision are screened preoperatively at day 0 (visit 1). Patients are enrolled given their ability to understand the extent and nature of the trial and their provision of written informed consent after detailed patient information. All inclusion criteria and no exclusion criteria must be fulfilled. Baseline data are collected during the screening/baseline visit. The duration of visit 1 will be approximately 25 min. Included patients are randomized during surgery (visit 2) after closure of the fascia in SST. Follow-up visits will be on postoperative days 5 to 7, 10 to 14 and 30 to 35 (visits 3–5) for evaluation of secondary endpoints (time expenditure approximately 15 min). In addition, 6, 12 and 24 months (visits 6–8) after surgery, patients are planned for follow-up to evaluate primary and secondary outcome parameters. The expenditure of time for each visit will be approximately 30 min per patient. An overview of trial visits and items captured during the trial visits is presented in Table 2 according to the guidelines of the Standard Protocol Items: Recommendations for Interventional Trials (SPIRIT) [29].

### Data management

All protocol-required information collected during the trial must be entered by the investigator, or designated representative, in an electronic case report form (eCRF) implemented in the Research Electronic Data Capture (REDCap™) system [31] (www.project-redcap.org). An explanation should be given for all missing data. The completed eCRF must be reviewed and signed by the investigator named in the trial protocol or by a designated sub-investigator. The Institute of Medical Biometry and Informatics of the University of Heidelberg (IMBI) is responsible for data management within the trial. To assure a safe and secure environment for any data acquired, data transmission is encrypted with Secure Sockets Layer (SSL) technology. Only authorized users are able to enter or edit data, and access is restricted to patients’ data in the respective centre. All changes to data are logged with a computerized timestamp in an audit trail. All data will be pseudonymized. Completeness, validity and plausibility of data will be checked in time of data entry (edit checks) and with the use of validating programs, which will generate queries. If no further corrections are to be made in the database, eCRF data will be locked. All data management procedures will be conducted according to written defined standard operating procedures (SOPs) of the IMBI that guarantee an efficient conduct complying with good clinical practice (GCP).

### Sample size calculation

**Fig. 1:**
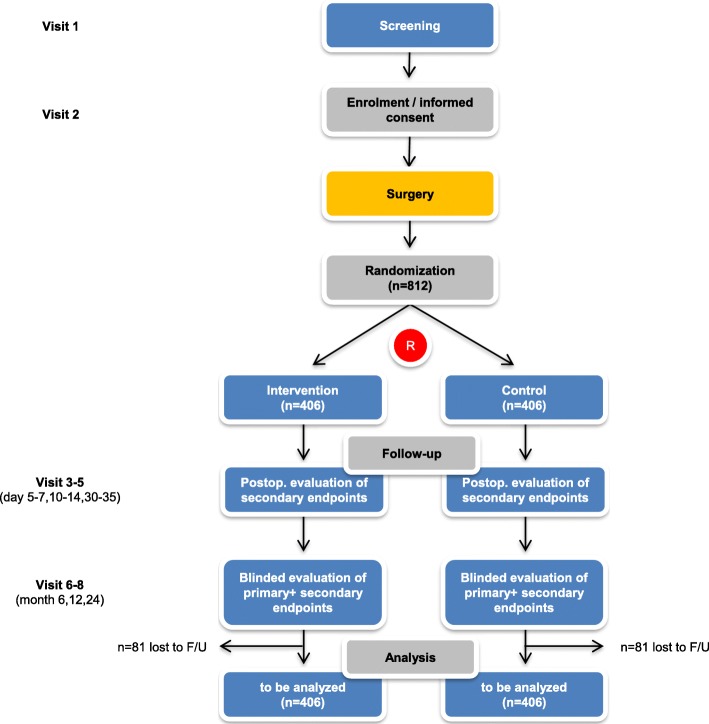
Trial flow chart

The sample size calculation is based on the primary efficacy endpoint (IH risk) within 24 months after surgery. Based on the assumption that the percentage of patients developing an IH after midline laparotomy in a general surgical population closed with the SST is approximately 15% for the control group, we hypothesize a reduction of 7% in the intervention arm based on previous RCTs [13, 20]. Consequently, a sample size per group of 325 patients is needed for the between-group comparison by the chi-squared test to achieve 80% power in detecting this difference in IH risk at a two-sided level of significance of 5%. It is assumed that using a two-level logistic regression model adjusting for the random factor centre and the fixed factor IH risk (low-risk vs. high-risk patients: BMI ≥ 27 and/or surgery for abdominal aortic aneurysm) [20] in the primary analysis will lead to less unexplained variance and thus to an increased power. Assuming a drop-out rate of up to 20% based on previous trials [1, 3, 10, 18], a total of 812 patients (406 per group) will be randomized in the study (Fig. 1). The potential occurrence of missing values for the primary outcome is partially addressed by the predefined multiple imputation strategy. Sample size calculation was performed using ADDPLAN v6.1.

### Analysis variables and statistical methods

The primary efficacy analysis will be based on the full analysis set (FAS) built according to the intention-to-treat (ITT) principle, thus reflecting the recommendations given in guidelines [32]. As a sensitivity analysis, an evaluation based on the per-protocol (PP) population (based on those patients without major protocol violation and excluding patients who receive a fascial closure not predefined in the randomization scheme) will be performed. The risk of IH will be analysed via a two-level binary logistic regression model including the fixed factors treatment group and IH risk (high vs. low), the latter being deemed as the most important confounder by far and being also used for stratification in the randomization procedure, and the random factor centre. Confounding by other less important prognostic and predictive factors can assumed to be controlled by the randomized study design. The model will be fitted using a variance-components covariance matrix. The level of significance is set to 5% (two-sided). All secondary outcomes will be evaluated descriptively, and descriptive *p* values will be reported together with 95% CIs for the corresponding effects.

### Further analyses

Interim analyses during the trial are not predefined or planned, but depending on the frequency of SAEs in both groups, safety analyses can be performed as recommended by the independent Data Safety Monitoring Board (DSMB). Further sensitivity analyses will be performed with the PP set, and the results will be compared with those of the ITT analysis. Moreover, for missing data in the ITT population set, further sensitivity analyses will be conducted by a worst-case scenario for the intervention, a minimal and a maximal IH risk imputation and by another alternative method of dealing with missing data as described by Higgins et al. [33]. Furthermore, a time-to-event analysis for the outcome “time from randomization to occurrence of IH” will be performed in the ITT population according to Kaplan-Meier. Additionally, prespecified subgroup analyses will be performed in the ITT population for the risk of incisional hernias in the subgroups of different types of surgery (colorectal, small bowel, hepatobiliary-pancreatic, upper GI (oesophageal and gastric), vascular, others), patients who are adipose vs. non-adipose and the presence or absence of neoadjuvant therapy, previous laparotomy or chronic obstructive pulmonary disease. All secondary outcomes will be evaluated descriptively, and descriptive *p* values will be reported together with 95% CIs for the corresponding effects.

### Safety analysis

The assessment of safety will be based on the frequency of SAEs in both groups, which will be analysed via descriptive statistical methods in the study population. For comparisons of frequencies between groups, the chi-squared test will be used. All analyses will be done using SAS version 9.4 or higher.

### Clinical data monitoring

Clinical monitoring will be performed by independent monitors of the Study Centre of the German Surgical Society (SDGC) according to its standard operating procedures in line with the ICH-GCP guideline (E6) [34]. A risk-based monitoring strategy will be conducted based on patient safety, patient rights, protocol adherence and data. The frequency of monitoring visits will be determined depending on recruitment numbers and individual performance of each centre based on feedback from project and data management.

### Premature closure of the trial

The trial may be prematurely closed by the coordinating investigator in consultation with the Steering Committee including the responsible biometrician. If termination of the trial becomes necessary, the Steering Committee of the trial will discuss this issue with the independent DSMB. Similarly, the DSMB can recommend closing the trial based on the safety reports; however, the decision remains with the Steering Committee. Reasons that may necessitate termination of the trial include the incidence or severity of SAEs, morbidity or complications in this trial that indicate a potential health hazard caused by the study treatment. Furthermore, the trial should be terminated if it appears that patients’ enrolment is unsatisfactory with respect to quality and/or quantity or data recording is severely inaccurate and/or incomplete. Another case in which termination of the trial becomes necessary is if external evidence demands a termination of the trial.

## Discussion

Despite the rise of laparoscopic surgery, open abdominal surgery by a midline laparotomy is still the most performed approach in abdominal surgery today [35]. Regardless of improvements in the surgical techniques of abdominal wall closure, IH incidence remains high and causes substantial morbidity and costs [24]. In the USA approximately 348,000 IH repairs leading to more than US$3.2 billion in healthcare expenditure are performed annually [36]. Similar per capita numbers have been reported in Germany, where more than 51,000 IH repairs are performed each year, making it one of the most frequently performed operations [37]. Total costs for IH repair were estimated to be 6451 € per patient in France [38]. Thus, reducing the IH risk by 5% was calculated to result in an annual cost savings of 4 million € [38]. Furthermore, IH-related reduction of the HRQoL is an important patient-reported outcome, as has been shown in recent trials [2].

Primary prevention of IH is of utmost importance, since recurrence and re-recurrence risks reach 40% [39, 40] and a considerable decrease of HRQoL [41]. Thus, prevention of IHs would have a significant impact on the patient’s well-being and the whole healthcare system by reducing complications, avoiding additional interventions and increasing the HRQoL of affected patients.

Among earlier techniques to optimize the abdominal wall closure and therefore reduce the occurrence of IHs, the SST and OMA are the two most recent and promising ones. So far there has been no RCT combining these two techniques of abdominal wall closure. The HULC trial will be the first RCT to fill this gap of evidence and will combine the usage of SST and the prophylactic mesh augmentation. The strengths of the HULC trial will be its randomized and blinded study design and the adhesion to the most recent evidence of abdominal wall closure regarding the control and intervention techniques. The HULC trial will be a multicentre trial including 12 high-volume centres in abdominal surgery to minimize selection bias. All centres will be trained to standardize the surgical techniques and outcome assessment as much as possible, leading to an expected low performance bias. Furthermore, as recommended recently [42], the HULC trial will perform blinding as far as practicable by blinding patients and outcome assessors to reduce performance and detection bias. For the control and intervention group of the HULC trial, the most promising techniques of recent studies will be used, including a slowly absorbable suture material in continuous suture technique in SST in both groups [1]. The intervention group will receive an additional non-absorbable mesh in the onlay position, as absorbable meshes have failed to show a reduction in IH risk [43] in contrast to non-absorbable ones, and the technique of OMA has been identified as superior to SMA recently [24]. For safety reasons regarding the development of SSI after receiving the OMA treatment in the intervention group, the HULC trial will perform continuous follow-up visits including the evaluation of SSI during the first year after surgery according to the recommendations of the CDC [25].

As earlier trials [6, 9] have shown an increase of IH risk even after 12 months, and a minimum follow-up period of 24 months has been proposed by the EHS for future trials, the HULC trial will follow this recommendation. The trial will include a large sample size with an adequate drop-out rate measured by a properly conducted sample size calculation based on the reliable results of earlier trials. The HULC trial will enable an adequate risk-benefit assessment due to secondary endpoints including all relevant intervention-related adverse events. It will also include an extensive HRQoL assessment as a secondary outcome, as the reduction in quality of life through IHs has been shown to be of great importance for patients [2].

In summary, the results of the HULC trial will influence future guidelines and surgical practice concerning abdominal wall closure.

### Trial status

This manuscript was written according to the most current version of the study protocol (version 1.1, last updated on June 25, 2019). Recruitment of patients for the HULC trial will start in August 2019. The clinical phase of the trial (last patient out) is expected to be completed in in August 2023.

## Supplementary information

**Additional file 1.** SPIRIT 2013 checklist: recommended items to address in a clinical trial protocol and related documents.

## Data Availability

After completion of the trial, the data obtained by the trial will be summarized and analysed according to this protocol and hereafter published in a peer-reviewed journal to be accessible by any healthcare professional, participant or the public. An anonymized minimal data set underlying the results of the trial will be made available upon publication of the final results as a supplement in line with national and international data protection rules.

## Abbreviations

ASA: American Society of Anesthesiologists
CDC: Centers for Disease Control and Prevention of Surgical Site Infections
DFG: German Research Foundation
DSMB: Data Safety Monitoring Board
eCRF: Electronic case report form
EHS: European Hernia Society
FAS: Full analysis set
GCP: Good clinical practice
HRQoL: Health-related quality of life
IEC: Independent ethics committee
IH: Incisional hernia
IMBI: Institute of Medical Biometry and Informatics of the University of Heidelberg
ITT: Intention-to-treat
OMA: Onlay mesh augmentation
PP: Per-protocol
RCT: Randomized controlled trial
SAE: Serious adverse event
SDGC: Study Centre of the German Surgical Society
SL: Suture length
SOP: Standard operating procedure
SPIRIT: Standard Protocol Items: Recommendations for Interventional Trials
SSI: Surgical site infection
SSL: Secure Sockets Layer
SST: Small stitches technique
WL: Wound length
